# Revealing common disease mechanisms shared by tumors of different tissues of origin through semantic representation of genomic alterations and topic modeling

**DOI:** 10.1186/s12864-017-3494-z

**Published:** 2017-03-14

**Authors:** Vicky Chen, John Paisley, Xinghua Lu

**Affiliations:** 1 Department of Biomedical Informatics, University of Pittsburgh, 5607 Baum Blvd, Suite 500, Pittsburgh, PA 15206 USA; 2 Department of Electrical Engineering, Columbia University, 500 W. 120th St., Suite 1300, New York, NY 10027 USA

**Keywords:** Cancer, Topic modeling, Semantic representation, Cancer genomics, Disease mechanisms

## Abstract

**Background:**

Cancer is a complex disease driven by somatic genomic alterations (SGAs) that perturb signaling pathways and consequently cellular function. Identifying patterns of pathway perturbations would provide insights into common disease mechanisms shared among tumors, which is important for guiding treatment and predicting outcome. However, identifying perturbed pathways is challenging, because different tumors can have the same perturbed pathways that are perturbed by different SGAs. Here, we designed novel semantic representations that capture the functional similarity of distinct SGAs perturbing a common pathway in different tumors. Combining this representation with topic modeling would allow us to identify patterns in altered signaling pathways.

**Results:**

We represented each gene with a vector of words describing its function, and we represented the SGAs of a tumor as a text document by pooling the words representing individual SGAs. We applied the nested hierarchical Dirichlet process (nHDP) model to a collection of tumors of 5 cancer types from TCGA. We identified topics (consisting of co-occurring words) representing the common functional themes of different SGAs. Tumors were clustered based on their topic associations, such that each cluster consists of tumors sharing common functional themes. The resulting clusters contained mixtures of cancer types, which indicates that different cancer types can share disease mechanisms. Survival analysis based on the clusters revealed significant differences in survival among the tumors of the same cancer type that were assigned to different clusters.

**Conclusions:**

The results indicate that applying topic modeling to semantic representations of tumors identifies patterns in the combinations of altered functional pathways in cancer.

## Background

Cancer is a complex disease involving multiple hallmark processes [1, 2], and aberrations in these processes are caused by SGAs that perturb pathways regulating these processes. Different combinations of pathways lead to heterogeneous oncogenic behaviors of cancer cells, which impact patient outcomes and response to treatment. Identification of patterns of pathway perturbations can reveal common disease mechanisms shared by a tumor subtype and such information can guide targeted therapy.

Transcriptomic data have been widely used to reveal different cancer subtypes among tumors of the same tissue of origin, and such studies have identified many clinically relevant subtypes, which have significant prognostic value [3–11]. However, transcriptomics-based subtyping does not provide insight into the disease mechanisms underlying each subtype, that is, transcriptomics-based subtyping does not reveal the causative pathways underlying the development of subtypes. As such, such subtyping does not provide guidance for targeted therapy. Another limitation of transcriptomics-based subtyping is that tissue-specific gene expression prevents discovery of transcriptomic patterns across cancer types. Recent pan-cancer studies found that tumors are invariably clustered according to tissue of origins when using features that are related to transcriptomics [12, 13]. Therefore, studying common disease mechanism of cancers should be addressed from new perspectives.

In order to gain a better insight into cancer disease mechanisms, an alternative approach is to study patterns of SGAs that perturb signaling pathways, with the goal of identifying which combination of perturbed pathways underlies each of the subtypes. It can be hypothesized that each cancer subtype is likely driven by a specific combination of perturbed pathways, and identification of such common disease mechanisms would provide guidance for targeted therapy.

However, the direct use of SGA data to identify these signaling pathways is challenging. This is because pathways are composed of multiple genes, and in different tumors the same pathway can be perturbed by distinct SGAs affecting different members of the pathway. As such, two tumors sharing common pathway perturbations may exhibit completely different sets of SGAs, making it difficult to detect similarities between tumors. Thus individual tumors may present itself with different genomic alterations, while undergoing the same pathway perturbations [14]. This effect is amplified by the fact that multiple pathways need to be perturbed for cancer to develop. All of this results in highly heterogeneous mutation patterns in tumors with common pathway perturbations.

**Fig. 1:**
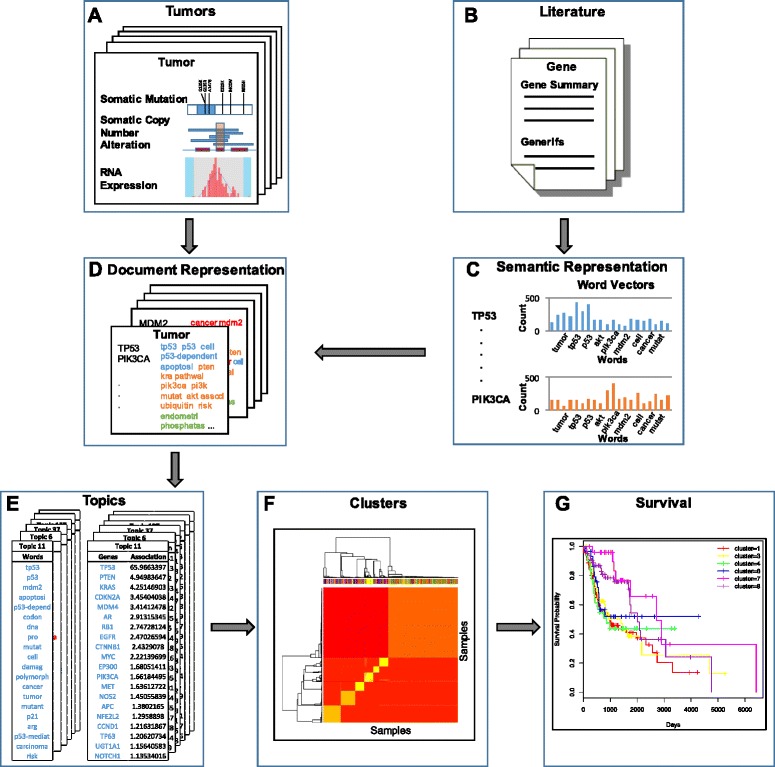
Conceptual Overview of Research. **a** Somatic mutations, copy number alteration and gene expression data for each tumor were collected. **b** GeneRIF and gene summaries associated with genes were collected. **c** The semantic data associated with each gene was processed to create a word vector representation (note the differences in the word frequency profile for different genes). **d** A document representation for each tumor was created by combining the word vectors of each SGA associated with the tumor. **e** The document representations were used as input for a hierarchical topic model, which identified topics associated with each tumor. **f** The tumors were represented in topic space, and clustering analysis was applied to group tumors with similar topic allocations. **g** These clusters were then used to perform survival analysis on tumors of the same cancer type

In order to tackle this problem, we have developed a novel semantic representation of genes that captures the similarity of functions of distinct genes. This representation would help us identify functionally related genes whose alterations result in similar changes in signaling pathways. We also chose to use topic modeling to identify patterns in these altered signaling pathways based on the semantic representations. The tumors were clustered based on these patterns, and survival analysis was performed on the results. The conceptual overview of our research is shown in Fig. 1.

## Methods

### Data processing

#### Cancer genomic data

Cancer somatic mutation data was downloaded (July, 2013) from The Cancer Genome Atlas (TCGA) and copy number variation and gene expression data was downloaded from The UCSC Cancer Genomics Browser [15, 16]. Data from five different cancer types was used: breast invasive carcinoma (BRCA), head and neck squamous cell carcinoma (HNSC), lung adenocarcinoma (LUAD), lung squamous cell carcinoma (LUSC), and ovarian serous cystadenocarcinoma (OV). The LUAD and LUSC data was combined into one large lung cancer (LUNG) dataset for processing.

#### Somatic mutations

PolyPhen-2 was used to determine which single-nucleotide-substitution mutations in a tumor had a potential effect on protein function, where each tumor was a different cancer tumor [17]. We considered a mutation event that was labeled either “possibly damaging” or “probably damaging” to be a functional mutation. The frame shift, nonsense, splice site, and multiple nucleotide mutations were considered functional mutations, because of their tendency to have a larger impact on protein function. This analysis was used to determine the functionally mutated genes for each tumor for each cancer type.

#### Copy number variation

We only considered the genes whose copy number variations resulted in an altered gene expression. In order to determine if the expression of a sample was altered, we first calculated the mean and variance of the samples with no copy number variation. These values were then used to calculate the probability of a gene to be differentially expressed using a one-tailed test on a normal distribution. If the probability fell below the threshold, then we considered the expression to be altered and kept the sample for further analysis. In this analysis, we only considered the instances where the gene was marked as +/− 2 in copy number, and a probability threshold of 0.01 was used. For each cancer type, we utilized the gene expression data that contained the most samples.

#### Combined data

The somatic mutation and copy number variation data were combined in order to get a more comprehensive view of the genes that are altered in each tumor. For each tumor, a gene that was either functionally mutated or affected by a copy number variation that resulted in an altered gene expression was considered an SGA event. In order to reduce the sizes of the datasets and decrease the chances of including passenger mutations, SGAs that occurred in less than 20 tumors were discarded.

The combined somatic mutation and copy number variation data resulted in datasets of the following sizes: BRCA with 779 samples and 15,517 genes; HNSC with 324 samples and 14,548 genes; LUAD with 398 samples and 11,851 genes; LUSC with 331 samples and 10,874 genes; and OV with 562 samples and 10,235 genes. This resulted in a dataset with 2,396 samples and 20,760 genes after combining all four cancer datasets, and 2,396 samples with 2,733 genes after applying the threshold.

### Semantic representation of SGAs

Function descriptions of each gene were obtained from GeneRIFs and gene summaries, which were downloaded from NCBI Gene on September 16, 2013. This text was preprocessed by removing stop words, tokenization, and Porter stemming [18]. Word vectors were created using GeneRIFs and gene summaries combined. The vocabulary size of the resulting word vectors were 57,035 words.

We calculated the term frequency-inverse document frequency (tf-idf) of each word to determine which words contained information pertinent to a gene. To do so, we treated the entire list of genes as one large corpus when calculating tf-idf score. Text from corresponding GeneRIFs and gene summary were pooled and represented as a document. The term frequency (tf) and document frequency (df) were calculated for each word for each gene document, with the term frequency being the number of times the word is associated with the document, and the document frequency being the number of gene documents the word is associated with. Using these values, we then calculated the tf-idf for a specific word with:

$$ \mathrm{tfidf}\left( w, d, D\right)=\mathrm{t}\mathrm{f}\left( w, d\right)*{ \log}_{10}\frac{\left| D\right|}{\mathrm{df}\left( w, D\right)} $$

where *w* represents the word, *d* is the tumor (or document), and *D* is the entire corpus. Thus |*D*| represents the total number of tumors. The cumulative tf-idf for each word was calculated by summing the tf-idf score across all documents. These cumulative tf-idf scores were used to limit the vocabulary size across the entire dataset. Only the 20,000 words with the highest cumulative tf-idf scores were included in the vocabulary.

### Semantic representation of SGAs and tumors

We created a word vector to represent each gene, consisting of words and their frequencies. A word vector was then created for each gene by including the 200 words with the highest tf-idf scores. Since a gene name and its aliases contains a large amount of information with respect to a gene, we set the tf-idf score for each gene name and alias in a word vector equal to the highest tf-idf score associated with that gene. In this way, an SGA event is not simply represented as a single gene name, which does not reflect the functional impact of the SGA, but rather it is represented by a word vector, such that the profile of words describing its function provides information of its functional impacts.

We further represented the SGAs observed in a tumor as a “text document” by pooling the word vectors associated with the SGAs. In this way, the functional themes of the SGAs are presented in the document, and tumors with similar pathway alterations are similar even though they may host quite different SGAs.

### Nested hierarchical dirichlet process

The nested hierarchical Dirichlet process (nHDP) is a hierarchical topic model [19], which uses Bayesian nonparametric prior to model the covariance of topics in a training corpus. nHDP represents the relations among topics using a tree, in which a node represents a topic and a path in a tree indicates that the topics on the path have a high tendency to co-occur in documents. When modeling the topics present in a text document, nHDP allows each document to access the entire tree [19] (considering all possible topics) and places a high probability on multiple paths. The nHDP algorithm was applied to a corpus of text documents representing tumors, and it returned a topic matrix, which defined the probability that a word is associated with a topic, and a document-topic distribution matrix, which defined how the words in a document are distributed among the topics. We used the parameter value *β*_0_ = 0.01, and we define the maximal level of the tree to be 3 and initialized the branching factor for a node at different levels (from root to leaf) to 10, 5, and 3. The nHDP algorithm was run 10 times to generate 10 different topic models for each dataset. The model that had the highest cumulative document likelihood was selected as the best-fitting topic model for further analysis.

### Mapping SGAs to topics

Since the topics in our setting reflect the functions that are repeatedly perturbed by SGAs among all tumors, it would be interesting to know which SGAs are associated with each functional theme. However, the nHDP model only captures the association of words with topics. Further calculations were needed to determine the SGAs associated with each topic. Utilizing the topic-to-document association and topic-to-word association matrices generated by the topic model, we represent the strength of association of an SGA with respect to a topic using *p*(*g*|*t*), which is calculated as follows:

$$ p\left( g\Big| t\right)\propto {\displaystyle {\sum}_d}{\displaystyle {\sum}_w} count\left( w\Big| g\right)* p\left( w\Big| t\right)* p\left( t\Big| d\right) $$

where *count*(*w*|*g*) is the word count for the word *w* in the word vector associated with the gene *g*; *p*(*w*|*t*) is the conditional probability of a word *w* given a topic *t*; *p*(*t*|*d*) is the probability that a word is assigned to topic *t* in document *d*.

### Clustering tumors

In order to find the tumors that share common disease mechanisms, we represented a tumor either as a vector spanning the SGA space, or as a vector spanning the topic space. We then performed consensus clustering to group the tumors. We used partitioning around medoids (PAM) as the base-line clustering method. For cluster sizes 4–6, the algorithm was run with 10 repetitions on the SGA space representations; for cluster sizes 4–10, the algorithm was run with 20 repetitions on the topic space representations. Consensus clustering was performed using the clusterCons package version 1.0 in R [20].

### Visualization of tumor clusters

In addition to consensus clustering, we also visualized the tumors (documents) in order to see how clearly our topic model was able to separate the different samples. The t-Distributed Stochastic Neighbor Embedding (t-SNE) technique of dimensionality reduction was used to plot the points in a two-dimensional space [21]. We used the Matlab implementation downloaded from http://homepage.tudelft.nl/19j49/t-SNE.html.

### Calculating cluster to topic associations

The proportion of samples (documents) in a cluster associated with each topic was calculated to see how topic associations vary between different clusters. In order to determine which documents are associated with each topic, the proportion of words from each document associated with each topic was calculated. Any topic that was associated with at least 0.01 of the words in a document was considered to be associated with the document. This threshold was used to remove associations that are the result of noise. We then obtained the proportion of documents in each cluster that are associated with each topic.

### Survival analysis

We performed survival analyses to evaluate the clinical impact of subtyping the tumors based on clustering. Tumors of the same cancer type were separated into subsets based on the clustering results obtained previously. Survival data for the tumors were obtained from the clinical data available on TCGA. The analysis was performed twice for each cancer type: once using all tumors, and once after excluding all clusters that contained less than 25 samples. We used the survival package version 2.38.3 in R to conduct the analysis [22, 23].

## Results

### Semantic representation reveals functional similarity among genes

We first examined whether word vectors representing SGAs highlight the similarities and differences between two genes. A subset of words and their tf-idf scores from the word vectors of three genes are shown as examples in Table 1. *TP53* is a tumor suppressor that is involved in apoptosis and DNA repair, and *MDM2* is a proto-oncogene that inhibits *TP53*. As expected, the word vector representing these two genes share common words and profiles. On the other hand, the *TTN* gene encodes for a protein that is important in muscles, which shows quite a different word profile.

**Table 1:** Subset of words from word vectors for three different genes

TP53		MDM2		TTN	
Word	Tf-Idf	Word	Tf-Idf	Word	Tf-Idf
p53	4,084	hdm2	629	ttn	88
tp53	4,084	mdm2	629	titin	88
cell	1,443	hdmx	629	domain	31
cancer	890	p53	363	pevk	18
express	887	cell	150	region	17
mutat	788	cancer	136	protein	16
activ	683	associ	117	muscl	15
gene	615	regul	113	mutat	15
associ	614	activ	97	structur	14
protein	602	express	95	elast	12
tumor	563	snp309	95	mechan	12
regul	505	protein	90	heart	11
carcinoma	465	risk	83	interact	11
role	456	suggest	76	molecular	11
apoptosi	418	result	74	express	10
result	405	tumor	73	stiff	10
function	397	polymorph	70	cardiomyopathi	10
pathwai	387	ubiquitin	69	studi	10
dna	384	interact	66	famili	10
suggest	371	degrad	66	sarcomer	10

### nHDP identifies biologically sensible topics

The goal of using topic modeling is to capture recurrent semantic themes (defined by a set of commonly co-occurring words) that exist in text documents representing SGAs in a collection of tumors. Presence of such a theme in the corpus usually is due to the repeated occurrence of SGAs in tumors that share a common functional description (although containing different genes). We trained 10 nHDP models and selected the one that fit the input data the best. The model contains 205 topics that were allocated to at least one document.

**Fig. 2:**
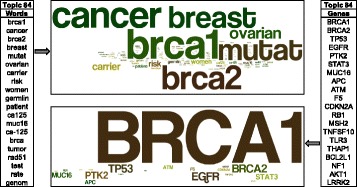
Example Topic Associations. The topic words (top 20) and SGAs for topic #84 is shown. On the left are the words associated with the topic, and on the right are the SGAs that are commonly associated with the cluster. In the center are the word cloud representations of the words and genes, on the top and bottom respectively

We inspected the words that constitute the topics and the SGAs associated with them, and an example topic is shown in Fig. 2. It is clear this topic is related to *BRCA1/2* genes and their relationship to cancer, particularly breast and ovarian cancers. The main function of *BRCA1/2* is related to DNA repair, and we found words related to DNA repair in the topic but they did not rank high enough to be shown in the figure, which only shows the top 20 words. Interestingly, *RAD51* gene, another DNA-repair gene that binds with *BRCA2* [24] and is regulated by *BRCA1* [25], is ranked high, indicating that the nHDP model was able to capture the DNA-repair theme. Similarly, three genes that are strongly associated with this topic are *BRCA1*, *BRCA2* and *TP53*; all are related to DNA repair, and they commonly occur in breast and ovarian cancers.

### Semantic representation of tumors reveals shared subtypes across cancer types

**Fig. 3:**
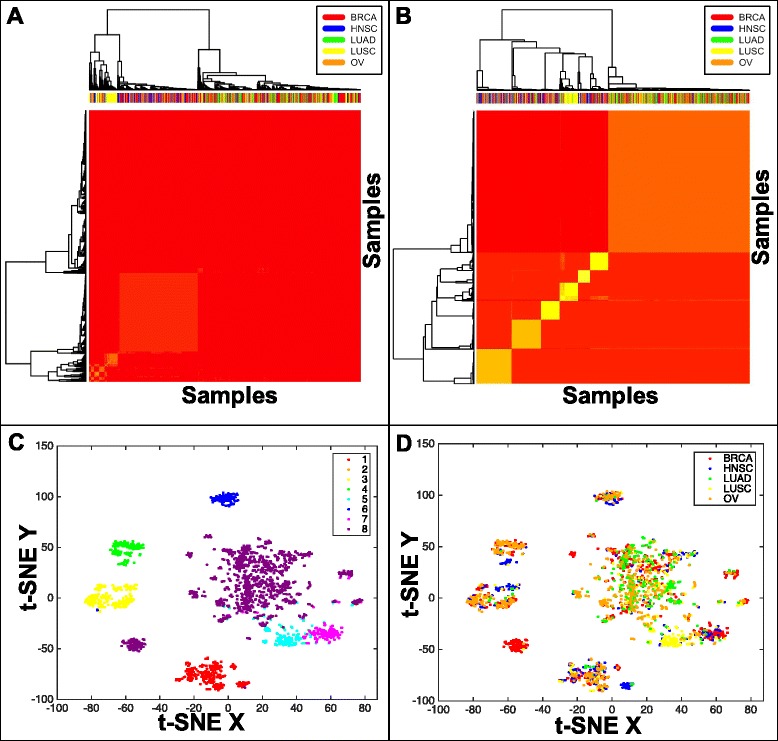
Tumor Clustering and Visualization. Both **a** and **b** are the consensus clustering results of all of the tumors. **a** Consensus clustering of tumors based on their genomic alterations. **b** Consensus clustering of tumors based on their topic associations. **c** and **d** are the results of t-SNE embedding and visualization of tumors represented in topic space. In Panel **c**, the tumors are labeled based on the clustering results seen in **b**; in Panel **d**, the tumors are labeled based on their cancer type

As stated previously, a main goal of this study is to use genomic alteration data (SGAs) to reveal common disease mechanisms shared among tumors (within or across cancer types). We found that clustering in the SGA space did not result in clean clusters for any of the cluster sizes (Fig. 3a). This result is expected because the heterogeneity of SGAs among tumors prevent the clustering algorithm from finding the similarity among tumors. In comparison, representing tumors in the semantic space–each tumor is represented as a vector spanning the topic space–revealed clear-cut clusters using either consensus clustering or t-SNE projection (Fig. 3b and c). The clearer separation of clusters in the topic space indicates that the topics captured the recurrent semantic themes (potentially reflecting functions of perturbed pathways), thus enabling the clustering algorithm to detect the similarity of tumors sharing common themes. It is particularly interesting that the majority of clusters contains tumors from multiple cancer types, indicating that certain semantic themes are shared among the tumors from different cancer types.

### Distinct topic allocation patterns across clusters

**Fig. 4:**
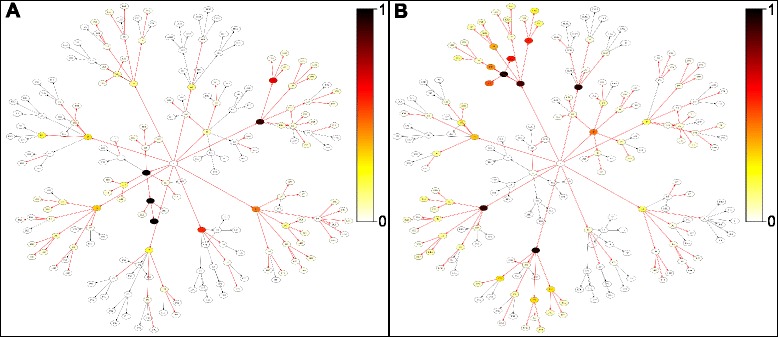
Graphical visualization of cluster-to-topic associations. The calculated degree of cluster-to-topic associations for two of the clusters using the clustering results seen in Fig. 3b. These visualizations show the structure of the topic tree, where each node represents a topic, and the different patterns of topics associated with individual clusters. The color scale denotes the proportion of tumors in a cluster associated with each topic, where white means that none of the tumors in the clusters are associated and black means that all of the tumors are associated with the topic. **a** The visualization for the topics associated with cluster 4. **b** The visualization for the topics associated with cluster 5

A key motivation of employing nHDP, instead of other probabilistic topic models such as the LDA model, is that nHDP not only detects recurrent themes but also, importantly, the covariance structure of topics. In other words, if a topic represents a pathway perturbed by SGAs, nHDP can capture the patterns of pathway perturbations. We examined and illustrated example topic allocation trees, which shows the proportions of samples in a cluster that are associated with each topic (Fig. 4). Apparently, the pattern of topic associations differed between clusters, and certain subtrees are strongly associated with one cluster but not the other. This implies that the combination of semantic (functional) themes, rather than the possession of unique functional themes, is what separates the different clusters. While we found that many topics close to the root would show up in multiple clusters, there are other more specific topics that are exclusive to one cluster. This was expected, because the topics that are close to the root in the hierarchy are more general functional themes and could be shared across clusters. However, the topics deep in the hierarchy are more specific and so should appear in fewer clusters.

### Survival analysis

**Fig. 5:**
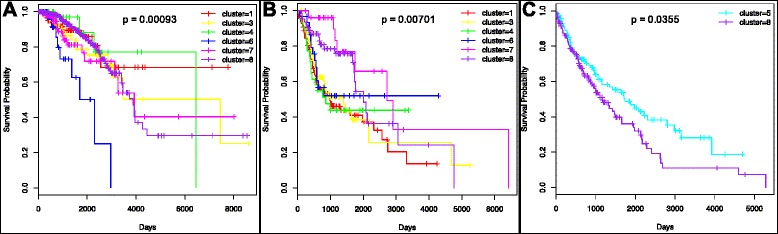
Survival analysis of tumors. The survival analysis curves calculated using only the clusters that contain at least 25 samples. Figs. **a**, **b**, and **c** correspond to cancer types BRCA, HNSC, and LUSC respectively

Assuming that different clusters consist of tumors sharing common disease mechanisms, we performed survival analysis to determine if such subtyping reveals clinical differences. Using the 8 clusters generated to group the tumors, we performed survival analysis on each of the different cancer types, where tumors were grouped according to their cluster id obtained from the consensus clustering analysis. Of the five cancer types, BRCA, HNSC, and LUSC were all found to be significant. This was true both when all samples and clusters were used, and when only the clusters containing at least 25 samples were used. The resulting survival curves can be seen in Fig. 5. These results indicate that semantic representation and clustering revealed cancer subtypes that have significantly different tumors with biologically different features, which were identified using their topic associations.

## Discussion

In this study, we investigated the utility of semantic representation and topic modeling for identifying patterns in signaling pathway perturbations in different tumors. Our results show that semantic representation of SGAs makes it possible to detect the functional similarity of different genes, which in turn enabled nHDP to detect recurrent patterns of pathway perturbation. Interestingly, this approach enabled us to identify cancer subtypes (clusters) consisting of tumors with quite diverse tissues of origin, which exhibit significantly different clinical outcomes (survival).

To our knowledge, this is a novel approach to studying common disease mechanisms using genomic alteration data. Our approach is the first to generate semantic representations to capture the functional information of tumors. We conjecture that the existence of topics in this new representation is due to recurrent SGAs that perturb genes involved in a common biological process or pathway. As such, one can further hypothesize that the presence of a topic in a tumor represents that a specific pathway is perturbed in the tumor. Following the same vein of thinking, one can hypothesize that tumors within a cluster identified in this study share a common disease mechanism, i.e., they share a particular pattern of pathway perturbation. Further in-depth analysis of topics and associated SGAs is needed to examine if such a hypothesis is supported by the results. If proved to be the case, our finding can potentially guide therapy targeting specific combination of pathways.

This study also has its limitations. Semantic data is limited by the amount and breadth of current knowledge regarding genes, so genes that are not well research or functions that have not been discovered would not be properly represented.

## Conclusion

Our research is the first time semantic representations are applied in this way to represent cancer samples, as well as the first use of a hierarchical topic model in this aspect of biomedical research. Applying topic modeling to the semantic representations of tumors made it possible to identify patterns of perturbed pathways in cancer tumors. This enabled the identification of cancer subtypes containing different tissues of origin that exhibit significantly different survival outcomes. If these subtypes are shown to share patterns of pathway perturbations, then these methods can potentially be used to guide targeted therapy of cancer.
